# A Rare Coexistence of Mesenteric Lymphangioma and Ulcerative Colitis, Obscuring the Right Cause of Symptoms

**DOI:** 10.7759/cureus.65977

**Published:** 2024-08-01

**Authors:** Akinori Sekioka, Yoko Shono, Tetsuo Ito, Kunihiko Tsuboi, Shuichi Ota

**Affiliations:** 1 Gastroenterological Surgery, Osaka Saiseikai-Noe Hospital, Osaka, JPN

**Keywords:** calprotectin fecal test, blood stool, inflammatory bowel disease, ulcerative colitis, mesenteric lymphangioma

## Abstract

Lymphangiomas are rare, cystic tumors representing congenital malformation of the lymphatic vessels. Mesenteric lymphangioma (ML) is a rare presentation of lymphangiomas. Misdiagnosis of ML can occur because of its rarity and resemblance to other entities. Ulcerative colitis (UC) is the most common type of inflammatory bowel disease (IBD), with an increasing incidence in pediatric populations. Here, we present a rare case of the coexistence of ML and UC. The uncommon radiological findings of ML can lead to overlooking UC; however, slight dissociation between clinical symptoms and radiological findings and the consequential decision to further investigations enabled us to reach an accurate diagnosis and avoid delaying the treatment of UC.

## Introduction

Lymphangiomas are rare, cystic tumors representing congenital malformation of lymphatic vessels. Mesenteric lymphangioma (ML) is a rare presentation of lymphangiomas, in less than 1% of the cases with lymphangiomas [1]. Ulcerative colitis (UC) is the most common type of inflammatory bowel disease (IBD), with an increasing incidence in pediatric populations [2]. The database of PubMed was searched for studies published up to July 2024, using the keywords “lymphangioma” and “ulcerative colitis” or “inflammatory bowel disease.” Based on the result, there was no previous report on the coexistence of ML and UC. Here, we report a case of the coexistence of ML and UC. The uncommon radiological findings of ML and the patient’s symptoms could obscure the right cause.

## Case presentation

A local clinic referred a 15-year-old boy to the emergency care department of our hospital during a weekend shift, suspecting acute perforated appendicitis. His symptoms were fever, abdominal pain, diarrhea continuing for the last three days, and blood stool for only one time. He had no history of other diseases; however, he had a family history of UC.

**Figure 1:**
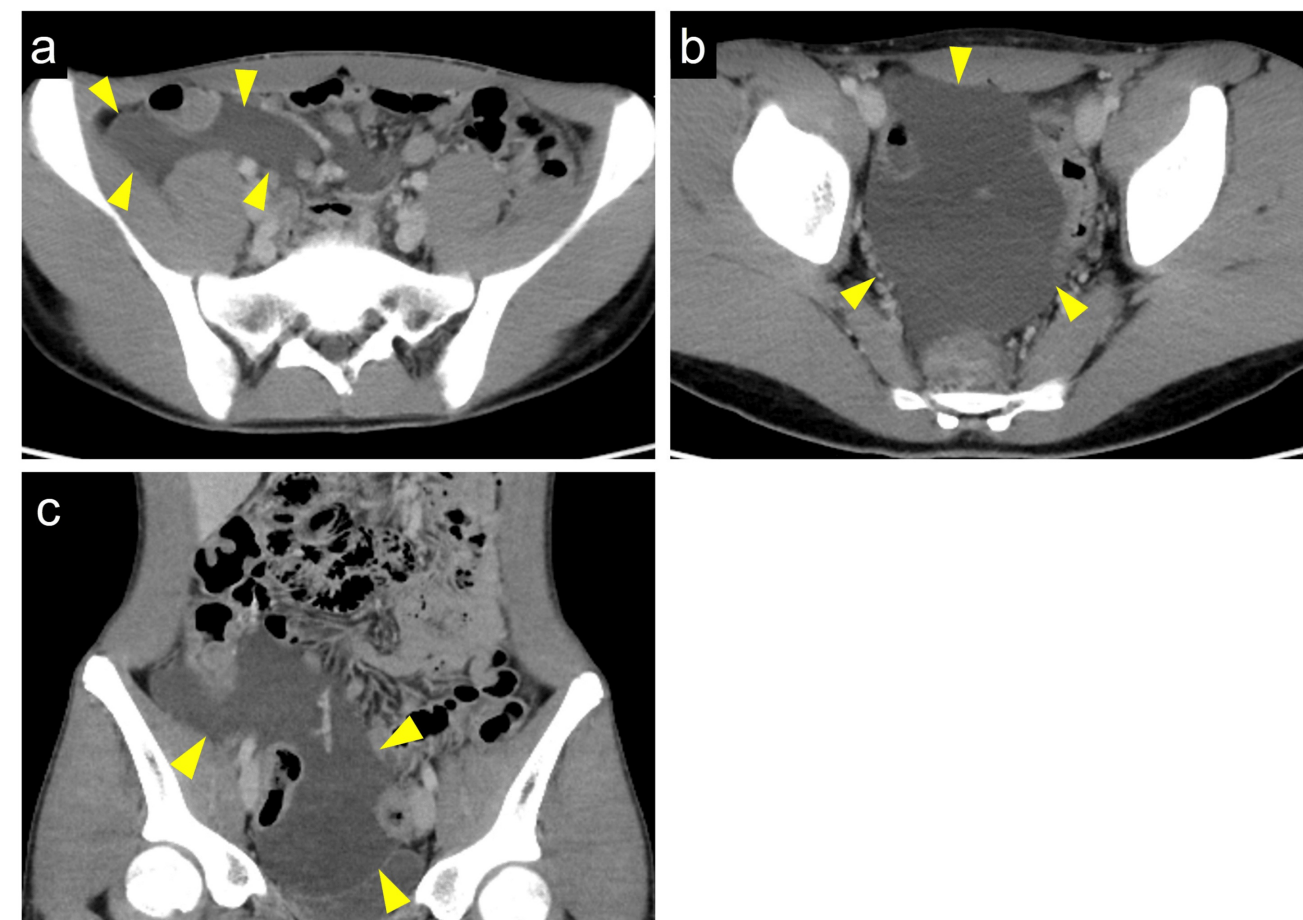
Contract-enhanced computed tomography (CT) showing mesenteric lymphangioma (a) The axial view of CT shows a fluidlike lesion in the right iliac fossa (arrowheads). (b) Another axial view of CT shows a fluidlike lesion in the pelvis (arrowheads). (c) The coronal view of CT shows the fluid-level lesion extending from the right iliac fossa to the pelvis (arrowheads)

**Figure 2:**
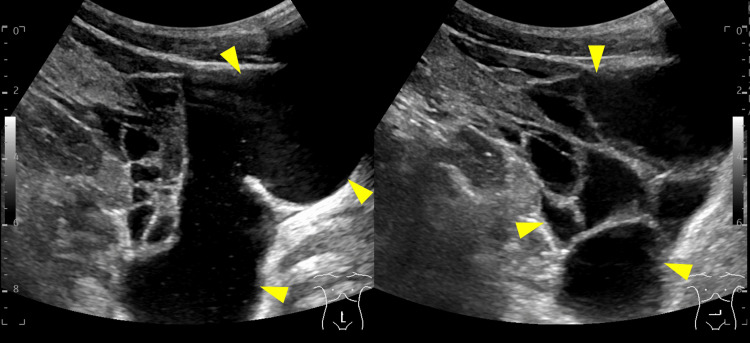
Transcutaneous abdominal echography The fluidlike space in computed tomography (CT) comprised multiple cystic lesions (arrowheads)

On general examination, his blood pressure was 147/76 mmHg, heart rate was 90 bpm, body temperature was 37.4℃, and peripheral oxygen saturation on room air was 98%. Abdominal examination showed mild tenderness at the right lower quadrant and no Blumberg’s sign. Laboratory test results demonstrated elevated inflammatory markers; white blood cell count of 4000/µl (normal value 3,500-8,000/µl), C-reactive protein of 1.5 mg/dl (normal value < 0.3 mg/dl), and procalcitonin of 0.16 ng/ml (normal value < 0.046 ng/ml). Computed tomography (CT) showed ascites around the right lower abdomen and pelvic space but did not detect the appendix (Figure 1a-1c). Since his symptoms were not severe, conservative treatment was initially started. In the following weekday shift, radiologists, gastroenterologists, and surgeons reviewed the CT findings. After the discussion, additional ultrasonography (US) was performed, and it diagnosed ML, presenting with multicystic low echoic space around the superior mesenteric vessels (Figure 2). There were no signs of intracystic hemorrhage and infection.

**Figure 3:**
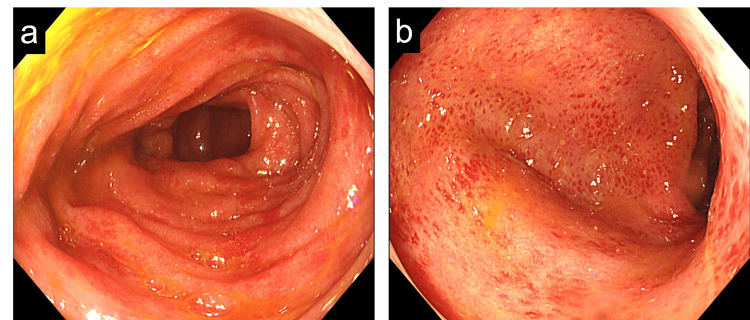
Colonoscopy showing diffuse erythema and edema in the total colonic mucosa, with loss of vascular pattern (a) At the transverse colon, (b) at the sigmoid colon

Since his symptoms were slightly incompatible with the clinical presentations of ML, different examinations were added; quantitative fecal immunochemical test was 1000 ng/ml (normal value < 100 ng/ml), and calprotectin fecal test was 134 mg/kg (normal value < 50 mg/kg). For further investigation, total colonoscopy was performed, revealing edematous and erythematous mucosa with friability in the total colon and a Matts score grade of 3 (Figure 3a-3b). The biopsy of colonic mucosa showed neutrophilic infiltrate and crypt abscesses without granulomas. Through these findings, we diagnosed a coexistence of ML and UC, and the latter one caused the patient’s symptoms. Lialda, a mesalazine, was introduced (3,600 mg/day) on day 7 post-admission, and the symptoms gradually improved. On day 14, he was discharged.

**Figure 4:**
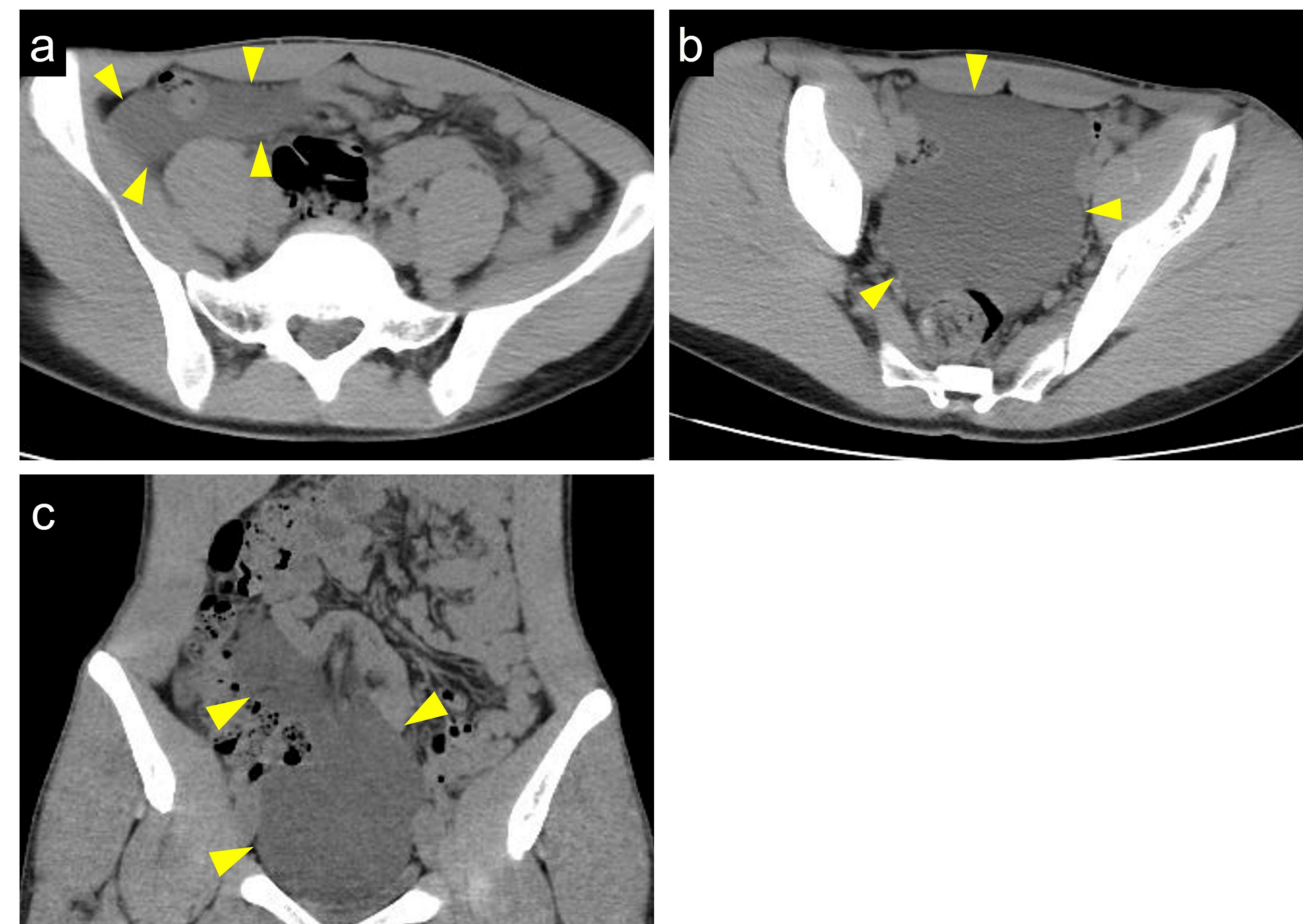
Plain computed tomography (CT) showing mesenteric lymphangioma with no remarkable change compared to two years ago (a) The axial view of CT shows the fluidlike lesion in the right iliac fossa (arrowheads). (b) Another axial view of CT shows the fluidlike lesion in pelvis (arrowheads). (c) The coronal view of CT shows the fluid-level lesion extending from the right iliac fossa to the pelvis (arrowheads)

After three months, the dose of mesalazine was tapered to 2,400 mg/day. On the follow-up colonoscopy after nine months from onset, mucosal inflammation improved. The follow-up CT after two years from onset showed no remarkable change in ML (Figure 4a-4c). There were no symptoms related to ML for this observation period.

## Discussion

Lymphangiomas are rare congenital malformations in the lymphatic systems, mainly found in childhood [1]. The common occurrence sites of lymphangiomas are the neck and axilla, while less than 5% of them occur in the abdomen. Abdominal lymphangiomas are frequently located in the mesentery, followed by the omentum, mesocolon, and retroperitoneum. Because of the rarity and the various clinical presentations of abdominal lymphangiomas, such as vomiting, diarrhea, constipation, abdominal distention, anemia, ascites, or lower gastrointestinal tract bleeding, it is often difficult to diagnose [3]. The diagnosis of lymphangiomas basically requires pathological evidence, while the recent guideline showed that the diagnosis can be confirmed by typical radiological findings of some modalities, such as US, CT, or magnetic resonance imaging [4].

UC is a common type of IBD, which is increasing in the total population, including children [2]. The early diagnosis and treatment of pediatric IBD is crucial because extensive bowels are more frequently involved and the symptoms exacerbate more rapidly than that of adult IBD. Although colonoscopy is essential for the diagnosis of IBD, there are some barriers to this investigation among pediatric patients, because the practical and emotional preparation for the examination is not easy for them [5,6].

In the present case, further investigations, such as fecal tests and subsequent colonoscopy, were performed despite radiological examinations revealing ML, because blood stool was a considerably rare symptom of ML. Most of the previous cases of gastrointestinal tract bleeding due to abdominal lymphangioma illustrated intraluminal lesions, such as ulcers or submucosal masses [7-9]. The present case did not show radiological findings suggestive of those lesions. Consequently, the calprotectin fecal test indicated gut inflammation and led to the decision to perform a colonoscopy, which was essential for the definitive diagnosis of IBD. Since the diagnostic delay in pediatric IBD is common and can increase the risk for complications and comorbidities [10], the present case’s decision to more precisely examine the cause of blood stool was useful to improve his outcome.

It is still debatable whether patients with scarcely symptomatic intraabdominal lymphangioma can be candidates for treatment. The guideline recommended that it is proposed to consider therapeutic intervention when the asymptomatic lesion tends to enlarge or has become symptomatic, because there is risk of treatment-related complications, such as recurrence, bowel obstruction, chylous ascites, embolism, hemorrhage, or wound infection [11]. If patients with asymptomatic abdominal lymphangioma select observation, periodic imaging studies are recommended.

According to the result of the search in PubMed database, this is a rare case report of the coexistence of ML and UC. Although it was a rare entity, early accurate diagnosis could shorten the length of untreated period and improve the prognosis.

## Conclusions

This is a rare case report of the coexistence of ML and UC. The rare radiological findings of ML can obscure the right cause of clinical symptoms. In the present case, the patient was examined very carefully and promptly. The slight dissociation between clinical symptoms and radiological findings and the consequential decision to further investigations enabled us to reach an accurate diagnosis and avoid delaying the treatment of UC.
